# Mental health, psychological wellbeing, and coping with stress by Ukrainian war refugees staying in Poland

**DOI:** 10.3389/fpubh.2025.1731764

**Published:** 2026-01-22

**Authors:** Agata Ewa Chudzicka-Czupała, Nina Zhou, Andrew Tay, Soon-Kiat Chiang, Monika Capińska, Vira Pomyluiko, Barbara Ostafińska-Molik, Marta Czupała, Marta Żywiołek-Szeja, Cheng-Fang Yen, Roger S. McIntyre, Roger C. Ho

**Affiliations:** 1 Interdisciplinary Center for Social Activity and Well-being Research, Faculty of Psychology, SWPS University, Katowice, Poland; 2 Yong Loo Lin School of Medicine, National University of Singapore, Singapore, Singapore; 3 Department of Psychological Medicine, Yong Loo Lin School of Medicine, National University of Singapore, Singapore, Singapore; 4 Institute for Health Innovation and Technology (iHealthtech), National University of Singapore, Singapore, Singapore; 5 ADRA Polska, Katowice, Poland; 6 Institute of Education, Laboratory of Health Pedagogy, Laboratory of Pedagogical Diagnoses, Jagiellonian University, Cracow, Poland; 7 Department of Psychiatry, Kaohsiung Medical University Hospital, Kaohsiung, Taiwan; 8 Department of Psychiatry, University of Toronto, Toronto, ON, Canada; 9 Department of Pharmacology, University of Toronto, Toronto, ON, Canada; 10 Division of Life Science, School of Science, Hong Kong University of Science and Technology, Hong Kong, Hong Kong

**Keywords:** coping mechanisms, DASS-21, forced migration, mental health, Poland, psychological wellbeing, Ukrainian refugees

## Abstract

**Background:**

The ongoing Russian-Ukrainian conflict has led to the large-scale displacement of over one million Ukrainian citizens, predominantly women and children, seeking protection in Poland. This necessitates a rigorous investigation into the factors associated with psychological conditions and adaptive coping mechanisms employed by this vulnerable population.

**Methods:**

This quantitative, online survey study comprised a sample of *N* = 290 adult participants (91.7% female, mean age 43.6 years). Due to the small number of male participants (*n* = 24, 8.3%), gender was not included as a variable in the analyses. Participants were recruited through social workers and psychologists working in Collective Accommodation Centers, and Integration Centers in Poland for Ukrainian refugees. Depression, Anxiety, and Stress symptoms were assessed using the DASS-21, psychological wellbeing with the PERMA-Profiler, and coping with stress strategies using the Brief-COPE.

**Results:**

Analysis revealed significantly elevated psychological distress among Ukrainian refugees in Poland. Multivariate regression models identified independent predictors of mental health, wellbeing and coping with stress. Older age, partnered status, and skills-matched employment are key predictors of depression. Higher education and partnered status are negative predictors of anxiety, and older age is a negative predictor of stress. Skills-matched employment emerged as a predictor of wellbeing, though only 23.5% of this highly educated sample held such positions. Access to information, receipt of psychological assistance, and current employment are predictors of problem-focused coping, while psychological assistance is a predictor of emotion-focused coping. Avoidant coping showed no significant model fit.

**Conclusion:**

The findings underscore the critical need for comprehensive, evidence-based mental health interventions and accessible psychosocial support for Ukrainian refugees. The results indicate the modifiable post-migration factors important for refugee adaptation. Policy priorities should include early mental health screening targeting younger adults and unpartnered individuals, facilitation of skills-matched employment, provision of clear information about legal rights and available services, and culturally sensitive interventions that promote adaptive coping mechanisms.

## 1 Introduction

In March 2022, the EU launched a temporary protection mechanism for refugees from Ukraine in light of the Russian-Ukrainian war. It was recently extended for more than four million Ukrainians until March 4, 2026 (1). Temporary protection is granted to the following people (as long as they lived in Ukraine on or before February 24, 2022), i.e., Ukrainian citizens and their family members, non-Ukrainians and stateless persons under international protection in Ukraine (e.g., refugees and persons under subsidiary protection) and their family members, non-Ukrainians with a permanent residence permit who cannot safely and permanently return to their country of origin (and who may also be under adequate protection in an EU country).

Temporary protection may also be granted, for example, to Ukrainian citizens who left Ukraine shortly before the war, as tourists or workers. To date, Poland's migration situation has been dominated by an increased influx of Ukrainian citizens. Three years after the Russian aggression against Ukraine, nearly one million Ukrainian citizens, primarily women and children, are currently benefiting from temporary protection in Poland. In total, 1.55 million people hold valid residence permits in this country.

Currently, 993,000 people are registered under this protection. Ukrainians are the largest group of foreigners in Poland, accounting for 78% of all foreign nationals settling in the country. Migrants from Ukraine living in Poland are mostly women (about 61%). Among adult refugees, women account for 78% of individuals. Additionally, 462,000 Ukrainian citizens hold valid temporary residence permits. The vast majority of these are issued in connection with employment. Meanwhile, 92,000 people hold permanent residence permits or EU long-term resident status. The territorial distribution of Ukrainians settling in Poland is characterized by concentration in provinces with large urban agglomerations. The most populous regions are: Mazowieckie Province−22% of all refugees, Dolnoślaskie Province−12%, Wielkopolskie Province−11%, Małopolskie Province−9%, and Slaskie Province−9% (2).

More than half of them are between the ages of 27 and 44. About 48% are people with higher education. Only 13% of them declare no knowledge of the Polish language. About 29% of refugees stay in Poland with a spouse/partner, and more than 40% of refugees live in Poland with underage children (3).

The increasing length of time refugees are staying in Poland and changes to regulations introduced in 2023, significantly limiting the possibility of free accommodation in organized housing offered by the Polish state, resulted in a further increase in the percentage of refugees renting accommodation on their own from 52 to 68%. In contrast, the percentage of refugees living in the organized housing decreased from 19 to 6%. Having a family significantly affects the housing situation of migrants (3).

The main source of income for migrants from Ukraine is work. In Poland, 94% of pre-war migrants and over 62% of refugees are employed. However, the nature of refugees' employment is less stable, as their work is most often casual, seasonal, and part-time. That is why the economic situation of refugees is more difficult than that of pre-war migrants. While the wages of pre-war migrants are largely above the minimum wage, almost half of refugees receive monthly amounts at the level of the minimum wage. They often face persistent economic uncertainty, which is a factor that increases their vulnerability to mental and medical disorders (3).

The psychological wellbeing and mental health of refugees may have deteriorated during emigration for various reasons, such as the outbreak of war and ongoing hostilities, the need to leave home, country, separation from family, the need to change jobs, place of residence, challenges of daily life (4). The study aimed to identify possible determinants of psychological wellbeing among immigrants, war refugees from Ukraine, living and adapting to life in Poland.

Promoting wellbeing has been identified as one of the United Nations' Sustainable Development Goals 2030 (5). The study substantiates the need to increase the psychological wellbeing of refugees from Ukraine and identifies areas where interventions should be made to effectively support refugees in coping with stress. The results of the study can have great applied value and serve as the basis for the design of interventions, adequate to the needs of those in need of psychological support.

Psychological wellbeing was studied in terms of the PERMA model (6). Seligman's PERMA framework (7) encompasses five fundamental elements that define an individual's psychological wellbeing, conceptualized as an integration of both hedonic and eudaimonic aspects. The PERMA acronym represents five core components: Positive Emotions, Engagement, Relationships, Meaning, and Achievement (6, 8). Within this theoretical approach, optimal functioning represents the pinnacle of wellbeing. The PERMA model suggests that this optimal state is characterized by an individual's capacity to experience positive emotional states, maintain life purpose, participate actively in engaging pursuits, cultivate meaningful interpersonal connections, and achieve personally significant accomplishments.

Uncertainty about the end of the war and the current geopolitical situation are significant stress factors that further increase the risk of mental health disorders and deterioration in the wellbeing. Research evidence indicates that even when residing in secure environments, refugees demonstrate elevated prevalence rates of depression, post-traumatic stress disorder (PTSD), and anxiety disorders compared to general populations unaffected by warfare (9–11). Several longitudinal investigations conducted among recently relocated refugees reveal that post-traumatic stress responses may persist and potentially intensify over extended periods (12–14).

Individual psychological responses to stress, including war-related trauma, exhibit considerable variation. This variation stems from differences in psychological sensitivity and resilience, mental fortitude, nervous system reactivity and endurance, along with other personal coping resources. When war-induced stress becomes sufficiently severe to overwhelm an individual's psychological coping mechanisms, acute stress disorder (ASD) may emerge following traumatic exposure, with post-traumatic stress disorder (PTSD) potentially developing subsequently (15, 16). Both ASD and PTSD represent psychiatric conditions that constitute responses to exceptionally distressing events or trauma that surpass a person's adaptive and coping capabilities. PTSD symptomatology typically manifests across three primary domains: intrusive experiences, avoidance behaviors, and hyperarousal states (17). The likelihood of developing ASD increases with advancing age and is commonly experienced among refugee populations. While PTSD is a significant mental health concern among refugee populations, the present study focuses on broader indicators of psychological distress, specifically symptoms of depression, anxiety, and stress, rather than trauma-specific symptomatology. In this context, understanding the general psychological wellbeing of refugees is essential.

While several studies have examined mental health among Ukrainian refugees in various European host countries, significant knowledge gaps remain. First, most existing studies concentrated primarily on symptom prevalence with limited investigation of positive wellbeing using comprehensive frameworks such as PERMA. Second, there is a notable lack of research examining the relationship between specific post-migration factors, such as access to information, employment matching skills, types of psychosocial support and mental health. Third, as displacement becomes prolonged (now exceeding 3 years), there is a growing need to understand refugees' longer-term adaptation processes. The present study addresses and partially fills these gaps by employing comprehensive assessment of mental health, wellbeing, and coping with stress, systematically examining post-migration factors, capturing prolonged displacement experiences (average 26.69 months), and providing evidence from Poland that can be used for comparative research.

Due to the exploratory nature of this study, no hypotheses were formulated. The aim was to find answers to the following research questions:

1. What is the mental health condition and psychological wellbeing of Ukrainian refugees staying in Poland?
2. Are the selected socio-demographic variables related to the experienced symptoms of depression, anxiety and stress and to the psychological wellbeing of refugees?
3. Are the selected socio-demographic variables related to the ways in which refugees cope with the stress of migration?

## 2 Materials and methods

### 2.1 Study design and population

This online survey study was conducted in Poland between June 2nd, 2024, and April 4th, 2025. Participants (290 adults, mostly women, *n* = 266; 91.72%) were recruited through survey links (Google Forms) distributed through the Facebook and Telegram platforms to social workers and psychologists working in centers that offer support to Ukrainian migrants (like Collective Accommodation Centers, Integration Centers for Foreigners). They electronically invited refugees to participate.

All ethical principles were observed at every step. The participants were informed of the study's objectives, assured of the anonymity and confidentiality of their responses, and advised of their right to withdraw from the study at any time without providing a reason or facing any consequences. Informed consent was obtained electronically, and no incentives were offered for participation. Interested candidates accessed the questionnaire by clicking on the “Agree to participate” button, after which they proceeded to provide their responses. Those who did not wish to participate could either opt out by selecting the “Not willing to participate” option or simply ignore the advertisement.

The present study protocol was approved by the Ethics Committee of SWPS University, Katowice, Poland (ethical committee approval number: WKEB103/10/2024).

### 2.2 Measures

Depression, Anxiety and Stress Scale–shortened version DASS-21 (Depression, Anxiety and Stress Scale (18)), with the time period extended to cover the last 3 months, in the Ukrainian version that was used. The Ukrainian version of the measure was adapted and validated in previous studies (4, 19). The scale consists of 21 items, seven for each subscale. Each item is rated on a four-point scale from 0 (never-did not apply to me at all) to 3 (almost always-applied to me very much, or most of the time). The higher the average scores obtained on the subscales, the greater the signs of emotional disturbance experienced.

We used the PERMA Profile, by Butler, and Kern (6), in Ukrainian language version by Ostafińska-Molik, Chudzicka-Czupała and Nadia Hapon (experimental version, 2023) for studying psychological wellbeing. The questionnaire measures five key dimensions of wellbeing: positive emotion (P), “In general, how often do you feel joyful?” engagement (E), “How often do you become absorbed in what you are doing?” relationships (R), “To what extent do you receive help and support from others when you need it?”, meaning (M), “In general, to what extent do you lead a purposeful and meaningful life?” and accomplishment (A), “How much of the time do you feel you are making progress toward accomplishing your goals?” (6).

Each of 15 items is rated on an 11-point Likert-type scale, where 0–never, 10—always, or 0–not at all, 10—completely, or 0–terrible, 10—excellent, with higher total scores indicating better subjective psychological wellbeing. The PERMA-Profiler has an acceptable model fit; internal consistency; test–retest reliability; and content, convergent, and divergent validity (6).

Another measure used was The Brief Coping Orientation to Problems Experienced (Brief-COPE) (20), used to assess ways of coping with stressful life events related to staying in Poland as a refugee. The Ukrainian version of the research tool was adapted and validated in previous studies (4, 19). The questionnaire consists of 28 items relating to 14 possible responses to negative life experiences, which can be categorized into three main coping styles: problem-focus, emotion-focus, and avoidance. The tool is derived from The Coping Orientation to Problems Experienced (COPE) inventory (20). Each item is rated on a four-point scale from 0 (I hardly ever do this) to 3 (I almost always do this).

Socio-demographic variables such as gender, age, education, nationality, refugee status, questions about received help and psychological support after arrival in Poland, professional work, and whether the respondent intends to remain in Poland were also controlled.

Supplementary Table 1 presents the internal consistency reliability coefficients—Cronbach's α and McDonald's ω—for the scales and subscales used in the study: DASS-21, PERMA, and Brief-COPE. All three DASS-21 subscales: Depression (α = 0.858; ω = 0.861), Anxiety (α = 0.876; ω = 0.874), and Stress (α = 0.872; ω = 0.874) exhibited high internal consistency, confirming their reliability for assessing psychological distress.

The PERMA subscales displayed good to excellent internal consistency, with Cronbach's α and McDonald's ω ranging from 0.773 to 0.885 and 0.805 to 0.886, respectively. The overall PERMA wellbeing index yielded very high reliability (α = 0.905; ω = 0.907). Additional indices, negative emotion (α = 0.763; ω = 0.750) and health (α/ω = 0.921)–also demonstrated strong internal consistency.

Among the Brief-COPE subscales, problem-focused coping demonstrated acceptable reliability (α/ω = 0.781), while emotion-focused coping showed lower internal consistency (α = 0.719; ω = 0.659), and avoidant coping fell below acceptable thresholds (α = 0.560; ω = 0.562), suggesting limited reliability. This raises concerns about the reliability of this subscale in the current sample and suggests that findings related to avoidant coping should be interpreted with caution.

Overall, the scales showed satisfactory psychometric properties, with the exception of the avoidant coping subscale, which may require cautious interpretation.

### 2.3 Sample

The study was conducted in refugee support centers located in Kraków and Katowice in southern Poland. These centers served Ukrainian refugees with varying economic circumstances and access to different forms of support. The online survey link was distributed through social workers and psychologists who shared it through their networks (Facebook, Telegram), potentially reaching refugees beyond those directly registered at the centers, including individuals who may not have visited the centers during the invitation period. The number of refugees accessing these centers was dynamic and fluctuating throughout the 10-month study period (June 2024–April 2025), as individuals entered and exited available services continuously. Additionally, a portion of questionnaires was distributed among refugees residing in collective accommodation centers, where living conditions were often significantly more challenging and socioeconomic vulnerability was likely higher. This mixed recruitment strategy suggests that the sample reflects a heterogeneous population rather than those exclusively in the most disadvantaged circumstances.

Supplementary Table 2 presents data on the sociodemographic characteristics of Ukrainian respondents (*n* = 290). The majority of respondents were women (91.72%, *n* = 266), while men constituted 8.28% (*n* = 24). One person (0.3%) chose not to answer the question regarding gender. The average age of the respondents was 43.62 years (SD = 12.53). Most participants had completed higher education at the master's level (52.8%, *n* = 153) or held a bachelor's degree (24.5%, *n* = 71). Vocational education (technical school) was reported by 5.9% of respondents (*n* = 17), while 4.1% (*n* = 12) had completed general secondary education. The average length of stay in Poland was 26.69 months (SD = 11.17). The data distribution indicates that most respondents had been living in Poland for more than 2 years. Most respondents lived in large cities (70.7%, *n* = 205). A significant portion of respondents rented apartments (70.0%, *n* = 203). Other groups included those living in group accommodation centers (13.8%, *n* = 40), renting a room (7.2%, *n* = 21), or staying with family or friends (9.0%, *n* = 26). Most respondents were married (59.7%, *n* = 173). A notable group consisted of divorced individuals (13.1%, *n* = 38) or those in informal relationships (10.0%, *n* = 29). Single individuals accounted for 11% (*n* = 32), while 5.9% (*n* = 17) of respondents were widows or widowers. Due to the small number of male participants in the sample, gender was not included as a variable in the analyses.

Supplementary Table 3 presents descriptive statistics for issues related to the stay of refugees from Ukraine in Poland. It presents detailed socio-economic data.

### 2.4 Statistical analysis

Descriptive statistics were calculated for socio-demographic characteristics, socio-economic integration, DASS-21, PERMA, and Brief-COPE. Categorical variables were presented as number of responses and percentage of response to the questions, which was calculated based on the number of respondents per response to the number of total responses to the question. Total scores and the respective subscale scores of DASS-21, PERMA, PERMA per item mean, Brief-COPE, and Brief-COPE per item, were calculated and expressed as mean and standard deviation. These scores assess the mental health condition and psychological wellbeing of the Ukrainian refugees staying in Poland.

Linear regression was then used to calculate the univariate associations between sociodemographic and socioeconomic characteristics against the total score of DASS-21 and the subscale scores of DASS-21, PERMA, and Brief-COPE. Subsequently, multi-linear regression was performed using variables that were significant as independent variables. This method was chosen to determine the variables that are still significantly related to the experienced symptoms of depression, anxiety, stress, psychological wellbeing of refugees, and coping levels with the stress of migration. Zero-order correlation was presented to show the direct correlation for the multi-linear regression. All tests were two-tailed with a significance level of *p* < 0.05. Statistical analysis was done using SPSS Statistic 29.

## 3 Results

Descriptive statistics for the scales and subscales are shown in Supplementary Table 4. The mean DASS-21 total score was 30.09 (SD = 13.56), reflecting moderate overall distress. Subscale means indicated that depression (M = 11.84, SD = 4.68) was slightly higher than stress (M = 9.51, SD = 5.01) and anxiety (M = 8.73, SD = 5.10). For the PERMA-Profiler, refugees reported moderate levels of positive emotion, engagement, relationships, meaning, and accomplishment, with scores ranging between 16.48 and 18.54. The composite PERMA wellbeing index averaged 15.79 (SD = 4.85), while negative emotion was elevated (M = 17.86, SD = 5.92). Health was rated moderately (M = 11.06, SD = 4.67), and loneliness was comparatively low (M = 5.17, SD = 2.89). To allow comparison with published PERMA benchmarks (scored on a 0–10 scale), raw domains (0–30) were divided by three to calculate per-item means. Both raw and per-item means are presented in the table. Regarding coping styles, emotion-focused coping (M = 16.88, SD = 5.73) was reported more frequently than problem-focused (M = 14.11, SD = 4.54) or avoidant coping (M = 8.75, SD = 3.54). To account for differences in the number of items across coping domains, we also report per-item mean scores (0–3) for the Brief-COPE. On this scale, problem-focused coping averaged 1.76 (SD = 0.57), emotion-focused coping 1.69 (SD = 0.57), and avoidant coping 1.46 (SD = 0.59). These values indicate that refugees more frequently endorsed problem- and emotion-focused strategies than avoidant coping.

### 3.1 Psychological distress

Linear regression analysis identified several factors associated with lower psychological distress (Table 1). Older age and being in a partnered relationship were linked to lower total DASS-21 scores (*p* < 0.05). Within the subscales, depression scores were significantly lower among older and partnered refugees (*p* < 0.05), as well as among those who had received general help and support, had access to information about their rights in Poland, and were employed in a job corresponding to their education or skills (all *p* < 0.05). For anxiety, both higher educational attainment (*p* < 0.01) and being partnered (*p* < 0.05) predicted lower scores, while older age was associated with significantly lower stress scores (*p* < 0.01). No other demographic or contextual variables showed significant associations after adjustment.

**Table 1:** Linear regression of DASS against demographic characteristics.

**Demographic characteristics**	**Total DASS**		**Depression**		**Anxiety**		**Stress**	
	***B*** **(SE)**	***p*** **-value**	***B*** **(SE)**	***p*** **-value**	***B*** **(SE)**	***p*** **-value**	***B*** **(SE)**	***p*** **-value**
Age (years)	−0.133 (0.063)	0.036^*^	−0.053 (0.023)	0.024^*^	−0.015 (0.024)	0.527	−0.065 (0.022)	0.003^**^
Education level	−1.109 (0.635)	0.082	−0.223 (0.235)	0.344	−0.631 (0.237)	0.008^**^	−0.255 (0.220)	0.248
Time in Poland (months)	−0.080 (0.071)	0.263	−0.032 (0.026)	0.229	−0.022 (0.027)	0.410	−0.026 (0.025)	0.292
Polish language proficiency	0.905 (0.828)	0.275	0.056 (0.306)	0.855	0.537 (0.311)	0.085	0.312 (0.286)	0.275
Place of living in Poland	0.379 (1.425)	0.790	−0.051 (0.526)	0.923	0.311 (0.536)	0.562	0.119 (0.492)	0.809
Housing	−0.557 (1.070)	0.603	−0.304 (0.395)	0.442	−0.130 (0.403)	0.746	−0.123 (0.369)	0.739
Marital status^a^	−3.933 (1.726)	0.023^*^	−1.631 (0.636)	0.011^*^	−1.358 (0.650)	0.038^*^	−0.944 (0.598)	0.116
Received help/support	−3.113 (1.715)	0.070	−1.382 (0.632)	0.03^*^	−0.943 (0.647)	0.146	−0.789 (0.593)	0.185
Received financial support	−1.844 (1.697)	0.278	−1.224 (0.624)	0.051	−0.515 (0.639)	0.421	−0.106 (0.587)	0.857
Received help finding accommodations	−1.615 (1.717)	0.348	−0.653 (0.634)	0.304	0.276 (0.647)	0.670	−0.687 (0.592)	0.247
Access to free Polish language course	0.301 (1.946)	0.877	−0.023 (0.719)	0.974	0.105 (0.732)	0.886	−0.382 (0.671)	0.570
Access to information about rights and obligations in Poland	−2.386 (1.644)	0.148	−1.247 (0.605)	0.04^*^	−0.611 (0.620)	0.325	−0.528 (0.569)	0.354
Received free psychological assistance	0.471 (1.687)	0.780	0.025 (0.623)	0.968	0.196 (0.635)	0.758	0.250 (0.582)	0.668
Received free support finding a job	−0.921 (1.934)	0.634	−0.535 (0.714)	0.454	−0.362 (0.728)	0.620	−0.025 (0.668)	0.970
Currently working	−0.029 (1.597)	0.986	−0.110 (0.590)	0.853	−0.316 (0.601)	0.600	0.397 (0.551)	0.472
Doing a job corresponding to education/skills	−2.757 (1.867)	0.141	−1.587 (0.686)	0.021^*^	−1.069 (0.702)	0.129	−0.101 (0.647)	0.876

^a^Recoded marital status (single, widowed, divorced = 1, married, in a relationship/cohabiting = 2). ^*^p < 0.05; ^**^p < 0.01; ^***^p < 0.001.

In the multi-linear regression model, where all significant variables were entered simultaneously (Table 2), the overall explanatory power was moderate. The model for total DASS-21 scores explained 38% of adjusted variance (Adjusted *R*^2^ = 0.38, *F* = 2.899, *p* < 0.01), with older age (*p* < 0.05) and partnered status (*p* < 0.05) remaining significant predictors. For depression specifically, the model demonstrated substantially stronger explanatory power (Adjusted *R*^2^ = 0.61, *F* = 4.146, *p* < 0.001), with older age (*p* < 0.01), partnered status (*p* < 0.05), and skills-matched employment (*p* < 0.05) retaining significance. The models for anxiety (Adjusted *R*^2^ = 0.26, *F* = 2.301, *p* < 0.05) and stress (Adjusted *R*^2^ = 0.27, *F* = 2.360, *p* < 0.05) showed weaker but statistically significant explanatory power, with higher education and partnered status predicting lower anxiety (*p* < 0.05), and older age predicting lower stress (*p* < 0.05). These findings highlight that while several sociodemographic and support-related variables appeared protective in the initial analyses, only a subset retained significance when examined together, with depression showing the strongest model performance and most robust predictor effects.

**Table 2:** Multi-linear regression of DASS against demographic characteristics.

**Demographic characteristics**	**Total DASS** ^**^		**Depression** ^***^		**Anxiety** ^*^		**Stress** ^*^	
	**(*****R***^**2**^ = **0.58)**		**(*****R***^**2**^ = **0.81)**		**(*****R***^**2**^ = **0.47)**		**(*****R***^**2**^ = **0.48)**	
	**(adjusted** ***R***^**2**^ = **0.38)**		**(adjusted** ***R***^**2**^ = **0.61)**		**(adjusted** ***R***^**2**^ = **0.26)**		**(adjusted** ***R***^**2**^ = **0.27)**	
	***F*****-statistic** = **2.899**		***F*****-statistic** = **4.146**		***F*****-statistic** = **2.301**		***F*****-statistic** = **2.360**	
	**Zero-order**	***p*** **-value**	**Zero-order**	***p*** **-value**	**Zero-order**	***p*** **-value**	**Zero-order**	***p*** **-value**
Age (years)	−0.123	0.030^*^	−0.133	0.010^*^	−0.037	0.544	−0.174	0.004^**^
Education level	−0.102	0.343	−0.056	0.883	−0.155	0.038^*^	−0.068	0.538
Marital status^a^	−0.133	0.018^*^	−0.149	0.005^**^	−0.122	0.039^*^	−0.093	0.070
Received help/support	−0.106	0.260	−0.128	0.228	−0.086	0.249	−0.078	0.472
Access to information about rights and obligations in Poland	−0.085	0.577	−0.121	0.269	−0.058	0.896	−0.055	0.764
Doing a job corresponding to education/skills	−0.087	0.182	−0.135	0.020^*^	−0.089	0.316	−0.009	0.763

^a^Recoded marital status (single, widowed, divorced = 1, married, in a relationship/cohabiting = 2). ^*^p < 0.05; ^**^p < 0.01; ^***^p < 0.001.

### 3.2 Psychological wellbeing

Linear regressions (Table 3) indicated that multiple contextual factors were associated with psychological wellbeing. Refugees who had received general help and support reported higher positive emotion and engagement, alongside lower negative emotion (all *p* < 0.05). Access to information about rights and obligations in Poland was linked to higher positive emotion, engagement, relationships, meaning, overall wellbeing, and health, as well as lower negative emotion (*p* < 0.05 to *p* < 0.01). Those who had received free psychological assistance reported greater positive emotion and meaning (*p* < 0.05), while refugees who had received free support in finding a job reported higher meaning and health (*p* < 0.05). Employment also mattered: being currently employed was associated with higher meaning, accomplishment, overall wellbeing, and health (*p* < 0.05 to *p* < 0.01), and being employed in a job corresponding to one's education or skills showed the broadest associations, with significantly higher positive emotion, engagement, relationships, meaning, accomplishment, overall wellbeing, and health, as well as higher loneliness scores (*p* < 0.01–0.001).

**Table 3:** Linear regression of PERMA against demographic characteristics.

**Demographic characteristics**	**Positive emotion**	**Engage-ment**	**Relation-ship**	**Meaning**	**Accomplish-ment**	**Overall**	**Negative emotion**	**Health**	**Loneliness**
	***B*** **(SE)**	***B*** **(SE)**	***B*** **(SE)**	***B*** **(SE)**	***B*** **(SE)**	***B*** **(SE)**	***B*** **(SE)**	***B*** **(SE)**	***B*** **(SE)**
Age (years)	−0.025 (0.028)	0.008 (0.030)	−0.049 (0.032)	0.012 (0.032)	0.000 (0.026)	−0.013 (0.023)	−0.134 (0.027)^***^	−0.14 (0.022)	−0.004 (0.014)
Education level	−0.184 (0.276)	−0.095 (0.300)	0.232 (0.325)	0.163 (0.321)	0.122 (0.263)	0.049 (0.228)	−0.314 (0.278)	0.586 (0.217)^**^	0.048 (0.136)
Time in Poland (months)	0.044 (0.031)	0.028 (0.034)	−0.008 (0.037)	0.059 (0.036)	0.004 (0.030)	0.020 (0.026)	−0.042 (0.031)	−0.008 (0.025)	0.027 (0.015)
Polish language proficiency	−0.245 (0.358)	−0.145 (0.390)	−0.047 (0.423)	−0.052 (0.417)	0.179 (0.342)	−0.055 (0.297)	0.329 (0.361)	−0.310 (0.285)	0.036 (0.177)
Have a place of living in Poland	0.374 (0.616)	−0.432 (0.670)	1.500 (0.722)^*^	0.834 (0.715)	0.249 (0.588)	0.523 (0.509)	0.790 (0.620)	0.544 (0.490)	0.230 (0.303)
Housing	0.398 (0.462)	−0.482 (0.502)	0.722 (0.544)	−0.133 (0.538)	−0.571 (0.440)	0.039 (0.383)	−0.541 (0.466)	0.404 (0.368)	0.219 (0.227)
Marital status^a^	0.964 (0.751)	0.801 (0.817)	3.351 (0.866)^***^	0.957 (0.873)	0.596 (0.718)	1.451 (0.617)^*^	−0.568 (0.759)	1.475 (0.594)^*^	0.460 (0.369)
Received help/support	1.533 (0.740)^*^	1.795 (0.804)^*^	1.329 (0.876)	1.532 (0.862)	0.077 (0.712)	1.032 (0.614)	−1.741 (0.746)^*^	0.869 (0.592)	0.685 (0.365)
Received financial support	1.370 (0.731)	1.191 (0.796)	0.280 (0.867)	1.534 (0.850)	0.942 (0.699)	0.820 (0.606)	−0.982 (0.740)	0.004 (0.586)	0.523 (0.360)
Received help finding accommodations	−0.154 (0.744)	0.513 (0.808)	0.657 (0.876)	−0.347 (0.864)	0.012 (0.710)	0.122 (0.615)	−1.374 (0.746)	0.384 (0.592)	−0.203 (0.366)
Access to free Polish language course	−0.067 (0.842)	−0.424 (0.915)	−0.097 (0.993)	−0.112 (0.978)	0.920 (0.801)	−0.319 (0.696)	−1.579 (0.844)	−0.750 (0.669)	−0.314 (0.414)
Access to information about rights and obligations in Poland	1.590 (0.708)^*^	1.669 (0.770)^*^	1.933 (0.834)^*^	2.282 (0.818)^**^	0.686 (0.680)	1.438 (0.584)^*^	−1.750 (0.713)^*^	1.563 (0.561)^**^	1.027 (0.346)
Received free psychological assistance	1.680 (0.723)^*^	0.837 (0.791)	1.105 (0.858)	1.976 (0.840)^*^	0.714 (0.695)	1.040 (0.600)	−0.567 (0.735)	0.857 (0.579)	0.741 (0.356)^*^
Received free support finding a job	1.152 (0.834)	1.057 (0.907)	0.917 (0.985)	1.903 (0.966)^*^	0.697 (0.798)	0.965 (0.689)	−0.099 (0.844)	1.390 (0.662)^*^	0.692 (0.410)
Currently working	0.910 (0.688)	1.225 (0.747)	1.282 (0.811)	1.940 (0.794)^*^	1.919 (0.649)^**^	1.267 (0.566)^*^	0.529 (0.696)	1.164 (0.546)^*^	0.578 (0.338)
Doing a job corresponding to education/skills	2.528 (0.797)^**^	2.337 (0.870)^**^	2.727 (0.942)^**^	4.399 (0.905)^***^	2.671 (0.757)^***^	2.603 (0.652)^***^	−0.666 (0.817)	2.305 (0.631)^***^	1.580 (0.388)^***^

^a^Recoded marital status (single, widowed, divorced = 1, married, in a relationship/cohabiting = 2). ^*^p < 0.05; ^**^p < 0.01; ^***^p < 0.001.

In the multi-linear regression model (Table 4), skills-matched employment and partnered status emerged as key predictors across multiple wellbeing dimensions, though the models showed varying levels of explanatory power. The strongest model performance was observed for relationships (Adjusted *R*^2^ = 0.78, *F* = 3.430, *p* < 0.001), where partnered status was the dominant predictor (*p* < 0.001). For overall wellbeing, the model explained substantial variance (Adjusted *R*^2^ = 0.69, *F* = 3.134, *p* < 0.001), with both partnered status (*p* < 0.01) and skills-matched employment (*p* < 0.05) as significant predictors. Negative emotion showed strong model fit (Adjusted *R*^2^ = 0.72, *F* = 3.808, *p* < 0.001), primarily predicted by older age (*p* < 0.001). Health demonstrated good explanatory power (Adjusted *R*^2^ = 0.72, *F* = 3.239, *p* < 0.001), with partnered status (*p* < 0.001) and skills-matched employment (*p* < 0.05) as key predictors.

**Table 4:** Multi-linear regression of PERMA against demographic characteristics.

**Demographic characteristics**	Positive emotion^**^	**Engage-ment**	Relation-ship^***^	Meaning^***^	Accomplish-ment^*^	Overall^***^	Negative emotion^***^	Health^***^	Loneliness^***^
	**(*R*^2^ = 0.84)**	**(*R*^2^ = 0.61)**	**(*R*^2^ = 0.91)**	**(*R*^2^ = 0.88)**	**(*R*^2^ = 0.61)**	**(*R*^2^ = 0.79)**	**(*R*^2^ = 0.89)**	**(*R*^2^ = 0.74)**	**(*R*^2^ = 0.79)**
	**(Adjusted *R*^2^ = 0.51)**	**(Adjusted *R*^2^ = 0.28)**	**(Adjusted *R*^2^ = 0.78)**	**(Adjusted *R*^2^ = 0.81)**	**(Adjusted *R*^2^ = 0.27)**	**(Adjusted *R*^2^ = 0.69)**	**(Adjusted *R*^2^ = 0.72)**	**(Adjusted *R*^2^ = 0.72)**	**(Adjusted *R*^2^ = 0.65)**
	***F*-statistic = 2.548**	***F*-statistic = 1.819**	***F*-statistic = 3.430**	***F*-statistic = 3.800**	***F*-statistic = 1.798**	***F*-statistic = 3.134**	***F*-statistic = 3.808**	***F*-statistic = 3.239**	***F*-statistic = 2.994**
	**Zero-order**	**Zero-order**	**Zero-order**	**Zero-order**	**Zero-order**	**Zero-order**	**Zero-order**	**Zero-order**	**Zero-order**
Age (years)	−0.052	0.016	−0.089	0.022	−0.001	−0.034	−0.284^***^	−0.038	−0.017
Education level	−0.039	−0.019	0.042	0.030	0.027	0.013	−0.066	0.157	0.021
Have a place of living in Poland	0.036	−0.038	0.122	0.069	0.025	0.061	0.075	0.065	0.045
Marital status^a^	0.075	0.058	0.222^***^	0.064	0.049	0.137^**^	−0.044	0.145^*^	0.073
Received help/support	0.121	0.130	0.089	0.104	0.006	0.099	−0.136	0.086	0.110
Access to information about rights and obligations in Poland	0.131	0.127	0.135	0.162	0.059	0.144	−0.143	0.162	0.172^*^
Received free psychological assistance	0.136^*^	0.062	0.076	0.137	0.060	0.102	−0.045	0.087	0.122
Received free support finding a job	0.081	0.068	0.055	0.115	0.051	0.082	−0.007	0.123	0.099
Currently working	0.078	0.096	0.093	0.142	0.172	0.131	0.045	0.125	0.100
Doing a job corresponding to education/skills	0.184^**^	0.156	0.168	0.275^***^	0.204^*^	0.229^**^	−0.048	0.210^*^	0.234^**^

^a^Recoded marital status (single, widowed, divorced = 1, married, in a relationship/cohabiting = 2). ^*^p < 0.05; ^**^p < 0.01; ^***^p < 0.001.

Loneliness also showed strong model performance (Adjusted *R*^2^ = 0.65, *F* = 2.994, *p* < 0.001), with skills-matched employment (*p* < 0.01) and access to information (*p* < 0.05) as significant predictors. The models for positive emotion (Adjusted *R*^2^ = 0.51, *F* = 2.548, *p* < 0.05) and meaning (Adjusted *R*^2^ = 0.81, *F* = 3.800, *p* < 0.001) demonstrated significance, with skills-matched employment predicting both outcomes (*p* < 0.05 for positive emotion; *p* < 0.001 for meaning), while psychological assistance additionally predicted positive emotion (*p* < 0.05). However, the models for engagement (Adjusted *R*^2^ = 0.28, *F* = 1.819, *p* > 0.05) and accomplishment (Adjusted *R*^2^ = 0.27, *F* = 1.798, *p* > 0.05) did not reach statistical significance, suggesting that the measured variables explain limited variance in these specific wellbeing dimensions. Overall, these findings indicate that partnered status and skills-matched employment are robust predictors of multiple wellbeing domains, particularly relationships and health, though not all PERMA dimensions are equally well-explained by the post-migration factors assessed.

### 3.3 Coping with stress strategies

Linear regression analyses (Table 5) indicated that several demographic and support-related variables were associated with coping strategies among refugees. Refugees who had received financial support reported more frequent use of both problem-focused and emotion-focused coping (*p* < 0.05). Access to information about rights in Poland was also associated with greater problem-focused coping (*p* < 0.01). Those who had received free psychological assistance demonstrated significantly higher levels of both problem-focused (*p* < 0.001) and emotion-focused coping (*p* < 0.01). Employment-related variables were also important: refugees who were currently working (*p* < 0.01) or employed in a job corresponding to their education or skills (*p* < 0.05) reported greater use of emotion-focused coping, while those who had received free support in finding a job showed a trend toward higher emotion-focused coping (*p* < 0.05).

**Table 5:** Linear regression of brief-COPE against demographic characteristics.

**Demographic characteristics**	**Problem-focused coping**		**Emotion-focused coping**		**Avoidant coping**	
	***B*** **(SE)**	***p*** **-value**	***B*** **(SE)**	***p*** **-value**	***B*** **(SE)**	***p*** **-value**
Age (years)	0.003 (0.021)	0.892	0.023 (0.027)	0.391	0.020 (0.017)	0.222
Education level	0.031 (0.214)	0.885	−0.191 (0.270)	0.480	−0.043 (0.166)	0.795
Time in Poland (months)	0.041 (0.024)	0.090	0.044 (0.030)	0.150	0.012 (0.019)	0.526
Polish language proficiency	−0.011 (0.278)	0.967	0.247 (0.350)	0.481	0.295 (0.216)	0.172
Place of living in Poland	0.141 (0.477)	0.768	0.556 (0.601)	0.356	−0.048 (0.371)	0.898
Housing	−0.190 (0.358)	0.596	0.111 (0.452)	0.807	−0.284 (0.278)	0.308
Marital status^a^	−0.860 (0.580)	0.139	−0.874 (0.734)	0.235	−0.814 (0.451)	0.072
Received help/support	1.111 (0.573)	0.054	0.845 (0.727)	0.246	−0.457 (0.449)	0.310
Received financial support	1.197 (0.564)	0.035^*^	1.485 (0.713)	0.038^*^	−0.072 (0.443)	0.871
Received help finding accommodations	0.544 (0.574)	0.345	0.059 (0.727)	0.936	−0.103 (0.448)	0.819
Access to free Polish language course	0.038 (0.651)	0.954	−0.313 (0.822)	0.704	0.279 (0.507)	0.582
Access to information about rights and obligations in Poland	1.504 (0.545)	0.006^**^	1.053 (0.695)	0.131	−0.351 (0.430)	0.414
Received free psychological assistance	1.960 (0.552)	< 0.001^***^	2.134 (0.702)	0.003^**^	0.005 (0.440)	0.991
Received free support finding a job	1.258 (0.643)	0.051	2.056 (0.809)	0.012^*^	0.317 (0.504)	0.530
Currently working	1.679 (0.525)	0.002^**^	1.262 (0.671)	0.061	0.024 (0.416)	0.954
Doing a job corresponding to education/skills	1.605 (0.620)	0.01^*^	1.238 (0.789)	0.118	−0.492 (0.488)	0.314

^a^Recoded marital status (single, widowed, divorced = 1, married, in a relationship/cohabiting = 2). ^*^p < 0.05; ^**^p < 0.01; ^***^p < 0.001.

In the multi-linear regression model (Table 6), differential patterns of predictive validity emerged across coping styles. The model for problem-focused coping demonstrated strong explanatory power (Adjusted *R*^2^ = 0.79, *F* = 5.158, *p* < 0.001), indicating that the measured post-migration factors substantially explain variance in adaptive problem-solving strategies. Within this model, access to information about rights (*p* < 0.05), receipt of free psychological assistance (*p* < 0.01), and current employment (*p* < 0.01) remained significant independent predictors. For emotion-focused coping, the model showed moderate but significant explanatory power (Adjusted *R*^2^ = 0.41, *F* = 3.034, *p* < 0.01), with psychological assistance (*p* < 0.05) as the sole remaining significant predictor.

**Table 6:** Multi-linear regression of brief-COPE against demographic characteristics.

**Demographic characteristics**	**Problem-focused coping** ^***^		**Emotion-focused coping** ^**^		**Avoidant coping**	
	**(*****R***^**2**^ = **0.89)**		**(*****R***^**2**^ = **0.60)**		**(*****R***^**2**^ = **0.09)**	
	**(Adjusted** ***R***^**2**^ = **0.79)**		**(Adjusted** ***R***^**2**^ = **0.41)**		**(Adjusted** ***R***^**2**^ = −**0.12)**	
	***F*****-statistic** = **5.158**		***F*****-statistic** = **3.034**		***F*****-statistic** = **0.429**	
	**Zero-order**	***p*** **-value**	**Zero-order**	***p*** **-value**	**Zero-order**	***p*** **-value**
Received financial support	0.124	0.291	0.122	0.194	−0.010	0.882
Access to information about rights and obligations in Poland	0.160	0.045^*^	0.089	0.486	−0.048	0.469
Received free psychological assistance	0.205	0.011^*^	0.176	0.045^*^	0.001	0.939
Received free support finding a job	0.115	0.624	0.148	0.137	0.037	0.432
Currently working	0.185	0.027^*^	0.110	0.242	0.003	0.540
Doing a job corresponding to education/skills	0.151	0.352	0.092	0.552	−0.059	0.245

^*^p < 0.05; ^**^p < 0.01; ^***^p < 0.001.

In contrast, the avoidant coping model showed no significant predictive validity (Adjusted *R*^2^ = −0.12, *F* = 0.429, *p* > 0.05), with no predictors retaining significance. The negative adjusted *R*^2^ value indicates that the model performs worse than a baseline intercept-only model, suggesting that the measured variables do not meaningfully explain variance in avoidant coping. This finding should be interpreted in light of the poor internal reliability of the avoidant coping subscale (α = 0.560) noted earlier, which limits the detectability of true associations. The strong performance of the problem-focused coping model (Adjusted *R*^2^ = 0.79) indicates that access to information, psychological support, and employment are powerful determinants of adaptive coping strategies, representing highly modifiable targets for intervention. The moderate performance for emotion-focused coping suggests that psychological support specifically facilitates emotional regulation strategies, though other unmeasured factors likely contribute substantially to this coping domain.

## 4 Discussion

The results of the study allows us to identify selected determinants of mental condition and psychological wellbeing, and make it possible to identify ways in which migrants in a refugee situation cope with stress. The key findings are presented across seven domains: (1) mental health (DASS-21), (2) wellbeing (PERMA-Profiler), (3) coping strategies (Brief-COPE), (4) core protective and risk factors across outcomes, (5) comparing the findings with existing research, (6), policy improvements, and (7) limitations and future study directions.

### 4.1 Mental health (DASS-21)

The DASS-21 yielded a mean total score of 30.09 among participants; however, the scale is typically interpreted using its individual subscales 18. Mean depression was 11.8, which is classified as mild depression, where 14 was the benchmark for moderate depression. Mean anxiety was 8.7, also in the mild anxiety range, with 10 as the benchmark for moderate anxiety. Mean stress was 9.5, which remains in the normal range with 15 as the benchmark for mild stress. The anxiety and depression levels are consistent with broader refugee populations, as war-displaced groups often show elevated rates of depression, anxiety, and stress compared to host communities (21). The above patterns align with current psychological theories on forced migration and trauma affecting mental health. Recent surveys indicate ~23% of Ukrainian refugees report mental health issues affecting daily functioning (22). The sample, composed primarily of middle-aged women, reflects a demographic consistently identified as more vulnerable to depression and anxiety in refugee populations, which may help explain the moderate levels of depressive symptoms observed (9). However, mean stress levels are in the normal range, possibly signaling the stability provided by Poland's integration mechanisms.

### 4.2 Wellbeing (PERMA-profiler)

The participants' mean per-item scores across the individual PERMA dimensions are as follows: Positive Emotion (5.49), Engagement (6.18), Relationships (5.82), Meaning (5.86), and Accomplishment (6.16). While there are no formally established clinical benchmarks, reference data from an international dataset of 31,966 participants reported higher mean per-item scores: Positive Emotion (6.69), Engagement (7.25), Relationships (6.9), Meaning (7.06), and Accomplishment (7.21) (6). Lower positive emotion likely reflects the pervasive grief, uncertainty, and cumulative trauma associated with forced displacement. The observed reduction in relationships is not unexpected, as many refugees are separated from their spouses, parents, or children and are compelled to reconstruct their social networks within unfamiliar cultural environments. This social fragmentation and family separation are recognized as significant indicators of distress among refugee populations (9). The reduction in meaning may originate from the sudden disruption of life plans, careers, and national identity brought about by armed conflict. Research indicates a strong correlation between a loss of purpose, an inability to envision the future, and the prevalence of depression and other symptoms of mental health disorders in displaced populations (15). Engagement and Accomplishment are reduced due to disrupted careers, education, and routines, as well as underemployment and legal uncertainty, which limit opportunities for progress and daily absorption (21).

Supplementary indices also indicated vulnerabilities, with overall wellbeing at 5.26, negative emotion at 5.95, health at 3.69, and loneliness at 5.17. International community benchmarks reported by Butler and Kern (6) indicate mean scores of 4.46 for Negative Emotion and 6.94 for Health. Health scores are reduced because refugees often face barriers to medical access, untreated chronic conditions, and the physical toll of displacement and prolonged stress. The worsened health conditions observed among refugees in our study are consistent with prior evidence on the long-term health outcomes of resettled refugee populations. Kumar et al. (23) found that refugees have significantly higher odds of chronic illnesses such as diabetes and hypertension compared to both host-country natives and non-refugee immigrants. This heightened risk is linked to cumulative stress exposures, disruption of healthcare access during displacement, and the socioeconomic challenges of resettlement.

### 4.3 Coping with stress strategies (brief-COPE)

While war and being a refugee may constitute significant stressors (4, 19, 24), the intensity of psychological responses that individuals experience is determined by their perceived capacity for managing stress and their subjective interpretation of the situation's significance, as outlined in Folkman and Lazarus's (25) transactional stress and coping framework. This cognitive evaluation process means that wellbeing enhancement strategies employed by different individuals may vary considerably. Moos (26) similarly highlights the significance of environmental factors and person-context interactions within his transactional model, emphasizing their combined influence on subjective cognitive assessment and coping mechanisms, as well as overall wellbeing and health outcomes. Evidence indicates that stress management approaches typically involve either problem-focused strategies, emotional regulation techniques, or avoidance-based responses designed to escape or withdraw from the stressor (25, 27, 28). It follows logically that individuals in different psychological conditions, displaying varied reactions to war-induced stress, experiencing different intensities of psychological symptoms (such as depression and anxiety), and manifesting varying degrees of stress manifestations (including intrusive thoughts, avoidance behaviors, and hypervigilance) will adopt distinct coping approaches.

On the Brief-COPE, mean per-item scores (1–4 scale) were 1.76 for problem-focused coping, 1.69 for emotion-focused coping, and 1.46 for avoidant coping. This pattern indicates that refugees in the sample relied most on problem-focused strategies (e.g., active coping, planning), followed by emotion-focused approaches (e.g., seeking emotional support, positive reinterpretation). Avoidant coping (e.g., denial, disengagement, substance use) was used least frequently. However, the low internal reliability of the avoidant coping subscale (α = 0.560) means these findings should be interpreted with caution, and we cannot draw strong conclusions about avoidant coping patterns in this population. These findings suggest that, despite exposure to significant stressors, participants tended to engage in adaptive coping methods rather than maladaptive avoidance. For Ukrainian refugees, this pattern is particularly meaningful, as displacement has often been accompanied by uncertainty, family separation, and challenges integrating into host societies. Problem-focused coping, such as actively seeking information, planning, and mobilizing resources, may help them regain a sense of control in otherwise unpredictable environments, thereby reducing feelings of helplessness. Emotion-focused strategies, including seeking social and emotional support, are also especially relevant given the strong communal and familial bonds in Ukrainian culture, which can provide resilience in exile. Conversely, the limited reliance on avoidance is encouraging, as avoidant coping in refugee populations has been consistently linked with higher levels of post-traumatic stress and depression (20). By leaning more heavily on adaptive coping, Ukrainian refugees may be better equipped to navigate displacement stress, which might have caused the normal stress levels in our study, maintain psychological wellbeing, and utilize host-country support systems more effectively.

### 4.4 Core protective and risk factors across outcomes. The role of socio-demographic and post-migration factors

Several factors consistently predicted better mental health and wellbeing, or constituted risk factors for mental health and wellbeing, across multiple outcomes, though the strength and significance of these associations varied considerably across models. Younger age was associated with higher depression, stress, and negative emotion. From a life-course perspective, experiencing forced migration during developmental transitions may be particularly destabilizing. This finding contrasts with Porter and Haslam's (29) meta-analysis, which found that child and adolescent refugees tended to have better relative outcomes than adults, while those aged 65 years or older had the poorest mental health outcomes. This could indicate a need for further research, as perhaps younger adults in the current Ukrainian refugee context face unique stressors, such as disrupted education, stalled career plans, and mismatch between life-stage expectations and realities of displacement, or the burden of providing for displaced family members, that contribute to heightened vulnerability. These pressures may create a mismatch between life stage expectations and the realities of displacement, amplifying depressive symptoms compared to older adults who may perceive their roles and responsibilities differently.

Age remained a significant predictor of depression in a regression model, providing robust evidence for this relationship. Younger age was likewise associated with higher stress scores, which is supported by current literature (30). This reflects evidence that younger refugees often experience elevated perceived stress from navigating host-country adaptation tasks such as schooling, labor market entry, and legal uncertainties. In contrast, older adults may draw on accumulated life experience and coping resources that buffer stress responses, even in displacement. Finally, younger refugees reported significantly higher Negative Emotion on the PERMA scale, indicating greater day-to-day experiences of anxiety, sadness, and anger. This is in line with broader findings that younger refugees often exhibit stronger affective reactivity to trauma exposure and post-migration stressors, whereas older refugees may display comparatively greater emotional regulation capacity (30–33). The adjusted model for negative emotion demonstrated strong explanatory power, with age as the primary significant predictor.

Marital or cohabiting status similarly buffered distress: partnered refugees reported lower DASS-21 scores (depression, anxiety, and overall scores), higher PERMA scores (relationship and overall scores), and physical health. Partnered status demonstrated particularly robust effects, emerging as a significant predictor in the depression model, the relationships model, the overall wellbeing model, and the health model. This underscores the protective role of close personal bonds in refugee mental health, echoing evidence that social support mitigates the impact of trauma and displacement (34, 35). Conversely, single or separated refugees may lack immediate support networks, potentially explaining their higher depression and anxiety. They may lack support during illness, potentially contributing to their poorer health outcomes.

Higher education was also significantly associated with lower anxiety and better self-rated health, indicating that educational attainment may act as a protective factor for both anxiety management and perceived physical wellbeing among refugees. However, in the anxiety model, education's effect was modest, suggesting that while protective, education's influence is tempered by other factors. Prior studies confirm that education is linked to improved mental health outcomes: for instance, higher educational attainment was associated with significantly lower odds of poor mental health in large-scale population studies (36), and among refugees, Syrian women with higher education reported lower psychological distress (37). This protective effect is likely explained in part by greater health literacy, which enables individuals to better understand and navigate healthcare systems, adhere to treatments, and adopt healthier behaviors more effectively (38). In addition, education cultivates stronger cognitive resources such as problem-solving, self-efficacy, and cognitive flexibility, which buffer anxiety in the face of uncertainty and support adaptive coping strategies.

Skills-matched employment showed significant associations with wellbeing outcomes, though its effects varied by domain. The employment variable predicted depression, positive emotion, meaning, overall wellbeing, health, and loneliness. Only 23.5% of our highly educated sample held skills-matched jobs, representing substantial underutilization. Employment carried the most significant associations, as simply being employed led to higher PERMA scores (meaning, accomplishment and overall score) and greater health. Research confirms employment quality not just status—predicts refugee wellbeing. The correlation with individuals who are employed in roles that align with their education or skills was even more pronounced, as these individuals exhibited reduced depression, higher PERMA scores across all domains, and improved health. Lai et al. (39) demonstrated that good quality employment is strongly associated with better physical and mental health outcomes among refugees. However, a positive correlation was observed between skilled employment and loneliness, suggesting that some refugees may have pursued employment as a coping mechanism for social isolation, utilizing work to re-establish structure and purpose in the absence of adequate relational support. Nevertheless, it demonstrates that the restoration of professional identity and competence may be as significant as income in influencing wellbeing. According to self-determination theory, fulfilling competence and autonomy needs is essential for wellbeing. A job that utilizes one's skills can restore agency which was stripped away by displacement. Empirical evidence confirms that decent work improves health and protects against depression, whereas unemployment worsens psychological distress (39). Work also provides structure, security, and social contact, which counteract the demoralization of idleness. At the same time, the association between skilled employment and higher loneliness is a reminder that work alone does not meet all needs; authentic social connectivity remains vital.

Access to information and services also demonstrated significant associations with psychological wellbeing outcomes. Greater access was linked to significantly lower depression (a negative association) and higher PERMA scores (Positive emotion, Engagement, Relationship, Meaning, and overall scores), alongside fewer negative emotions and better self-rated health. However, in adjusted models, information access retained significance primarily for and problem-focused coping, indicating that while information appears broadly beneficial in univariate analyses, its independent effects are most robust for enabling active problem-solving and may paradoxically relate to social isolation. The findings suggest that when refugees face uncertainty and lack of knowledge, this is associated with exacerbated depressive symptoms, whereas clear information on legal status, rights, and support options co-occurs with a sense of control. Refugees who reported being able to navigate systems (e.g., healthcare, schooling, legal aid) demonstrated greater wellbeing. Access to rights and information was also associated with greater use of problem-focused coping, suggesting that being more aware of their legal status and obligations may be related to the ability to actively tackle challenges arising during their resettlement in Poland. This is consistent with findings by Walther et al. (40), who showed that ambiguity and insecurity surrounding legal status were significant stressors for refugees, undermining mental health and integration. Their qualitative study highlighted how unclear bureaucratic processes and prolonged uncertainty fostered frustration, helplessness, and distress, whereas clarity and security in legal standing supported a stronger sense of belonging and psychological stability. Access to rights and information was also associated with greater use of problem-focused coping, suggesting that being more aware of their legal status and obligations might enable refugees to actively tackle challenges arising during their resettlement in Poland. This aligns with coping theory, which posits that individuals are more likely to engage in problem-solving strategies when they perceive stressors as manageable or within their control (25). In this sense, informational clarity may be perceived as a factor reducing uncertainty but also may empower refugees to respond more effectively to practical difficulties, thereby reinforcing adaptive coping and resilience. Although access to information was associated with lower distress and higher wellbeing, these findings should not be interpreted causally. Given the cross-sectional design, it is plausible that psychologically healthier refugees are more attentive to available resources, more proactive in seeking information, or more confident in navigating Polish institutions. Thus, the observed association may partly reflect differences in psychological functioning rather than the direct “effect” of information.

Finally, receiving psychosocial assistance and help reinforced the importance of mental health services in crises. The provision of essential assistance and support to refugees upon arrival, facilitating their adaptation to new circumstances, correlated with reduced rates of depression. It demonstrates that early intervention significantly mitigates the likelihood of depression during the critical resettlement phase. Meta-analytic evidence confirms that favorable post-migration conditions are strongly linked to improved mental health among refugees, with early access to accommodation, services, and social support predicting lower psychological distress (29).

Refugees who had access to free psychological support reported higher PERMA scores (positive emotion) and engaged in more problem-focused and emotion-focused coping mechanisms. However, they also reported higher loneliness, which may reflect that individuals are more likely to seek psychological support when they feel socially isolated. This dual pattern underscores the importance of interpreting service use both as a protective factor and as a marker of underlying vulnerability, highlighting the need for outreach models that reduce stigma and ensure access before distress becomes acute. Notably, the increased reliance on problem- and emotion-focused coping is itself adaptive: problem-focused coping enables refugees to take practical steps in addressing stressors, while emotion-focused coping helps regulate negative affect and maintain psychological equilibrium. Murray et al. (41) found that psychological and psychosocial programs led to reductions in depression, PTSD, and distress symptoms across diverse refugee populations.

### 4.5 The Polish context: comparing the findings with existing research

To our knowledge, this is the first study to systematically examine positive wellbeing among Ukrainian refugees using the PERMA framework, alongside mental health symptoms, post-migration protective factors (information access, skills-matched employment, types of social support), and coping strategies in the context of prolonged displacement (~27 months on average). Research on Ukrainian refugees in Poland conducted in April-May 2022 (21) found high levels of war trauma and stress among early arrivals, with younger refugees displaying particularly elevated distress, which aligns with our finding that younger adults experienced higher psychological distress. A more recent comparative study in Wrocław conducted October 2022-January 2023 (34) found that while 75% of Ukrainian refugees reported clinically relevant levels of psychological distress (primarily manifesting as somatic symptoms, anxiety, and insomnia), depressive symptom levels were comparable between Ukrainian refugees and Polish citizens. In the Wrocław study (34) researchers observed no significant differences in loneliness between Ukrainian refugees and Polish citizens, potentially reflecting Poland's favorable reception context and refugees' access to support networks. It contrasts with our finding that 76.5% of participants reported loneliness despite employment, suggesting that professional integration alone does not mitigate social isolation and that the passage of time (27 months on average in our study vs. ~10 months in the Wrocław study) may change and weaken initial social connections. Cross-national research comparing Ukrainian refugees in Poland and Hungary (42) demonstrated significant between-country differences, noting that Poland had been a favored destination for Ukrainian immigrants well before the war, allowing refugees to find substantial social support from the established Ukrainian diaspora, underscoring the critical role of host country reception policies and pre-existing community networks in facilitating adaptation. Studies examining mental health of internally displaced individuals and refugees in transit in Poland (43) as well as qualitative research on psychological resilience among Ukrainian refugee women (44) further demonstrate the complex interplay of trauma, coping resources, and contextual factors in shaping adaptation outcomes.

Our findings extend this body of Polish research by identifying the specific modifiable post-migration factors that may be important for refugees mental health, psychological wellbeing and coping with stress. Although our findings must be interpreted within Poland's specific policy and social context, they align with results from studies in other Ukrainian refugee host countries, revealing both commonalities and important differences that demonstrate how host-country factors shape refugee mental health (42, 45). The convergence across studies regarding high rates of psychological distress and the protective role of social support and marital status suggests these are universal aspects of the refugee experience, consistent with broader systematic reviews documenting elevated mental health risks among refugees resettling in high-income countries (29, 30). However, the divergence in findings, particularly regarding loneliness trajectories and the differential impact of employment, suggests that host country policies, pre-existing diaspora networks, labor market integration mechanisms, and cultural attitudes toward refugees significantly moderate outcomes (45). Our findings confirm the importance of selected factors essential for refugee adaptation, as described in the literature based on experiences from other countries such as Germany, Ireland, and the United Kingdom (46). Future research employing standardized measures across multiple host countries is needed to more rigorously determine which policies and practices most effectively support refugee integration and to understand how the passage of time differentially affects various aspects of adaptation in different national contexts.

### 4.6 Policy improvements

Our findings have direct implications for policy and practice. While our cross-sectional design precludes causal claims, the robust associations identified, combined with established evidence from intervention research and international best practices, suggest specific leverage points for improving refugee mental health and integration.

#### 4.6.1 Systematic mental health screening and early intervention

Our findings indicate that younger adults show significantly higher depression and stress scores, suggesting a need for targeted screening protocols. Early identification of mental health needs through systematic screening at the point of arrival is associated with better outcomes. Brief, validated screening tools should be integrated into standard health checks or registration procedures, especially for high-risk groups (younger adults, unpartnered individuals), to facilitate early provision of mental health support before conditions deteriorate. Scaling up culturally attuned services such as trauma-focused counseling and support groups is essential (46).

#### 4.6.2 Promoting social integration

Partnered status emerged as a strong predictor of wellbeing, yet 76.5% of participants reported loneliness, and even those in skills-matched employment reported social isolation. This underscores that professional reintegration alone does not address social needs. Programs fostering refugee-host community contact through community centers, cultural events, language classes, mentorship programs, and skilled volunteering opportunities can build social bonds and restore purpose. Research shows that volunteering reduces negative emotions and enhances self-esteem among displaced populations (46).

#### 4.6.3 Facilitating skills-matched employment

Skills-matched employment was significantly associated with depression, wellbeing, and health across multiple domains, yet only 23.5% of our highly educated sample held such positions. Priority recommendations include: streamlining credential recognition for Ukrainian professionals; establishing sector-specific programs with professional language training and mentorship; providing financial support for licensing exams and bridging education; and combating hiring discrimination. Access to continuing education should be scaled up to prevent deskilling (46).

#### 4.6.4 Ensuring access to information

Access to information about rights was a significant predictor of problem-focused coping, yet 37% of participants lacked adequate information upon arrival. While we cannot establish causality, information access is associated with enhanced active coping. Recommendations include multilingual guides and hotlines, community information hubs staffed by trained mediators, peer navigators from refugee backgrounds, and pictogram-based materials ensuring inclusivity for those with limited literacy.

#### 4.6.5 Implementation

The evidence-based recommendations align with international guidelines (UNHCR, IASC) and require coordination across government, NGOs, healthcare, employers, and education.

### 4.7 Limitations and future study directions

Although this study provides valuable insights into the psychological condition and coping strategies of Ukrainian refugees residing in Poland, several limitations should be acknowledged.

Our sampling strategy (online recruitment disseminated through social workers and psychologists in integration centers) likely introduced selection bias, limiting representativeness. Refugees who were more digitally literate, more engaged with support services, or more comfortable discussing psychological issues may be overrepresented. Conversely, individuals who were more isolated, severely distressed, or digitally excluded may be underrepresented. The high educational attainment in our sample (77.3% with bachelor's degree or higher) aligns with evidence that Ukrainian war refugees are highly educated population estimates range from 48% (3) to 73% (48), but likely remains inflated due to recruitment of service-engaged individuals.

A significant limitation concerns the gender distribution in our sample. The predominantly female composition (91.7%, *n* = 266) reflects the demographic reality of Ukrainian refugee populations, where women typically constitute 80%−90% of adult refugees due to mobilization restrictions on men aged 18–60. The small number of male participants (*n* = 24, 8.3%) severely limits the reliability and generalizability of gender comparisons. Consequently, gender was not included as a variable in our regression analyses, as the small male subsample would yield unreliable estimates and insufficient statistical power for meaningful comparisons. While this reflects the natural composition of this refugee population (47), it precludes examination of potential gender differences in how post-migration factors influence mental health, coping, and wellbeing. Future research should employ targeted recruitment strategies to ensure adequate male representation, enabling valid gender-comparative analyses. This might involve outreach through male-dominated employment sectors, partnership with Ukrainian cultural organizations serving men, and offering flexible survey completion options (offline, telephone) for populations less engaged with digital platforms.

A precise response rate could not be calculated due to dynamic refugee populations accessing centers throughout the 10-month study period, individuals accessing multiple services across centers, precluding determination of unique reachable individuals, and dissemination via social media networks reaching refugees beyond those formally registered. These factors indicate that the sample does not fully represent the broader Ukrainian refugee population in Poland, and findings should be interpreted with this limitation in mind. To improve representativeness, future studies should complement online recruitment with offline, community-based outreach and targeted efforts to engage less digitally connected refugees.

Another important limitation is connected with the poor internal reliability of the avoidant coping subscale (Cronbach's α = 0.560, McDonald's ω = 0.562). This low reliability likely contributed to the weak explanatory power of the avoidant coping model and the absence of significant predictors in adjusted analyses. The reliability issues may reflect cultural differences in how avoidant coping is conceptualized among Ukrainian refugees, the heterogeneity of avoidant coping strategies (denial, disengagement, substance use, self-blame), or translation/adaptation issues. Given this limitation, findings related to avoidant coping should be interpreted with caution, and we cannot draw strong conclusions about predictors of avoidance-based coping in this population. Future studies should consider using alternative measures or qualitative approaches to better capture avoidant behaviors.

While several of our models demonstrated strong to moderate explanatory power, particularly problem-focused coping, relationships, negative emotion, health, overall wellbeing, loneliness, and depression, other outcomes showed substantially weaker or non-significant variance explained. Specifically, the models for stress, anxiety, engagement, accomplishment, and avoidant coping explained less than one-third of variance or failed to reach statistical significance, indicating substantial unexplained variance and suggesting important unmeasured factors.

Potentially relevant unmeasured factors include pre-displacement trauma exposure, personality traits (neuroticism, conscientiousness, resilience), individual psychological resources (e.g., resiliency, self-efficacy, intrinsic motivation), quality (not just presence) of social support networks, cultural fit with host society and religious values, perceived discrimination and experiences of xenophobia, socioeconomic status and material losses, ongoing family separation and safety concerns for relatives in Ukraine, detailed employment characteristics beyond skills-match (e.g., job security, workplace discrimination, income adequacy, work-life balance), meaningful volunteer opportunities, and access to education or skill development programs. Future research should incorporate these variables to better understand the full constellation of factors influencing refugee adaptation.

The cross-sectional design precludes causal inferences despite the strong associations and high explanatory power observed. While our models identified robust predictors explaining explaining considerable variance in problem-focused coping (79%), relationships (78%), and depression (61%), we cannot determine whether these factors directly cause improved outcomes or whether refugees with better psychological resources are more likely to access employment, information, and support. Reverse causality remains plausible, as psychologically healthier refugees may be more proactive in seeking information, more successful in securing skills-matched employment, and more likely to engage with psychological services.

Longitudinal research is essential to establish temporal precedence and causal pathways, examine how relationships between predictors and outcomes evolve over time, identify critical periods when interventions would be most effective, determine whether initial levels of distress predict subsequent access to resources, and track changes in wellbeing as displacement becomes increasingly prolonged. Panel studies following refugees from arrival through long-term settlement would be particularly valuable for understanding adaptation trajectories and testing intervention effects.

## Data Availability

The raw data supporting the conclusions of this article will be made available by the authors, without undue reservation.
