# Supplementary material for: Mental health, psychological wellbeing, and coping with stress by Ukrainian war refugees staying in Poland
Source: Front Public Health. 2026 Jan 22;13:1731764. doi: 10.3389/fpubh.2025.1731764 (PMC12872490; doi:10.3389/fpubh.2025.1731764)
Supplement: Supplementary file 1 [file Table_1.docx]

Supplementary Material

# Supplementary Table 1. Reliability of individual scales and subscales

|  |  | Cronbach's α | McDonald's ω |
| --- | --- | --- | --- |
| DASS-21 | Depression | 0.858 | 0.861 |
|  | Anxiety | 0.876 | 0.874 |
|  | Stress | 0.872 | 0.874 |
| PERMA | Positive emotion: P | 0.885 | 0.886 |
|  | Engagement: E | 0.785 | 0.811 |
|  | Relationships: R | 0.813 | 0.815 |
|  | Meaning: M | 0.773 | 0.805 |
|  | Accomplishment: A | 0.822 | 0.825 |
|  | Overall Well-being PERMA | 0.905 | 0.907 |
|  | Negative emotion: N | 0.763 | 0.750 |
|  | Health: H | 0.921 | 0.921 |
| Brief-COPE | Problem-focused coping | 0.781 | 0.781 |
|  | Emotion-focused coping | 0.719 | 0.659 |
|  | Avoidant coping | 0.560 | 0.562 |

# Supplementary Table 2. Socio-demographic characteristics of the sample

| Items | Categories | Total (N=290) | % |
| --- | --- | --- | --- |
| Gender | Female | 266 | 91.72 |
|  | Male | 24 | 8.28 |
|  | I do not want to answer | 1 | 0.3 |
| Age (years) | Mean ± SD | 43.62± 12.53 |  |
| Previous education | Vocational education (school) | 17 | 5.9 |
|  | Professional higher education  (technical school) | 21 | 7.2 |
|  | General secondary (school) | 12 | 4.1 |
|  | Higher/Bachelor's degree | 71 | 24.5 |
|  | Higher/Master's degree | 153 | 52.8 |
|  | Candidate of Sciences / Doctor | 14 | 4.8 |
|  | Other | 2 | 0.7 |
| Time spent in Poland (month) | Mean ± SD | 26.69 ± 11.17 |  |
| Level of Polish language | Basic | 84 | 29.1 |
|  | Communicative | 119 | 41.2 |
|  | Fluent | 53 | 18.3 |
|  | Don't know | 33 | 11.4 |
| Place of living in Poland | Village | 13 | 4.5 |
|  | Small town | 72 | 24.8 |
|  | Big city | 205 | 70.7 |
| Housing (live with) | In a group accommodation center | 40 | 13.8 |
|  | Renting an flat | 203 | 70.0 |
|  | Rent a room | 21 | 7.2 |
|  | In family's/friends' apartment | 26 | 9.0 |
| Marital status | Single | 32 | 11.0 |
|  | Widower/widow | 17 | 5.9 |
|  | Married | 173 | 59.7 |
|  | Divorced | 38 | 13.1 |
|  | In a relationship/cohabiting | 29 | 10.0 |

# Supplementary Table 3. Socio-economic integration of migrants

| Question | Categories | Total (*N* = 290) | % |
| --- | --- | --- | --- |
| Did you receive the necessary help/support to adapt to the new conditions upon arrival? | Yes | 200 | 69.0 |
|  | No | 90 | 31.0 |
| Did you receive financial support? | Yes | 195 | 67.2 |
|  | No | 95 | 32.8 |
| Did you receive help in finding accommodation? | Yes | 91 | 31.4 |
|  | No | 199 | 68.6 |
| Did you have access to free Polish language courses? | Yes | 228 | 78.9 |
|  | No | 61 | 21.1 |
| Upon arrival, did you have access to reliable information about your rights and obligations in Poland? | Yes | 182 | 63.0 |
|  | No | 107 | 37.0 |
| Did you receive free psychological assistance? | Yes | 97 | 33.6 |
|  | No | 192 | 66.4 |
| Did you receive free support in finding a job? | Yes | 62 | 21.5 |
|  | No | 227 | 78.5 |
| Are you currently working? | Yes | 139 | 48.1 |
|  | No | 150 | 51.9 |
| Are you doing a job that corresponds to your education/skills? | Yes | 68 | 23.5 |
|  | No | 221 | 76.5 |
| Do you plan to stay in Poland? | I want to stay in Poland | 80 | 27.6 |
|  | I want to return to Ukraine | 64 | 22.1 |
|  | I would like to return, but I have to stay in Poland | 76 | 26.2 |
|  | I don't know | 70 | 24.1 |

# Supplementary Table 4. Scales and subscales scoring

|  |  | **Mean ± *SD*** |
| --- | --- | --- |
| **DASS-21** | Total | 30.09 ± 13.56 |
|  | Depression | 11.84 ± 4.68 |
|  | Anxiety | 8.73 ± 5.10 |
|  | Stress | 9.51 ± 5.01 |
| **PERMA (Raw score, 0-30)** | Positive emotion: P | 16.48 ± 5.87 |
|  | Engagement: E | 18.54 ± 6.38 |
|  | Relationships: R | 17.47 ± 6.92 |
|  | Meaning: M | 17.59 ± 6.82 |
|  | Accomplishment: A | 18.49 ± 5.60 |
|  | Overall Well-being PERMA | 15.79 ± 4.85 |
|  | Negative emotion: N | 17.86 ± 5.92 |
|  | Health: H | 11.06 ± 4.67 |
|  | Loneness: L | 5.17 ± 2.89 |
| **PERMA Per Item Mean (0-10)** | Positive emotion: P | 5.49 ± 1.96 |
|  | Engagement: E | 6.18 ± 2.13 |
|  | Relationships: R | 5.82 ± 2.31 |
|  | Meaning: M | 5.86 ± 2.27 |
|  | Accomplishment: A | 6.16 ± 1.87 |
|  | Overall Well-being PERMA | 5.26 ± 1.62 |
|  | Negative emotion: N | 5.95 ± 1.97 |
|  | Health: H | 3.69 ± 1.56 |
|  | Loneness: L | 5.17 ± 2.89 |
| **Brief-COPE** | Problem-focused coping | 14.11 ± 4.54 |
|  | Emotion-focused coping | 16.88 ± 5.73 |
|  | Avoidant coping | 8.75 ± 3.54 |
| **Brief-COPE Per Item (1-4)** | Problem-focused coping | 1.76 ± 0.57 |
|  | Emotion-focused coping | 1.69 ± 0.57 |
|  | Avoidant coping | 1.46 ± 0.59 |
